# Supplementary material for: Trait phenomenological control predicts experience of mirror synaesthesia and the rubber hand illusion
Source: Nat Commun. 2020 Sep 25;11:4853. doi: 10.1038/s41467-020-18591-6 (PMC7519080; doi:10.1038/s41467-020-18591-6)
Supplement: Supplementary file 1 — Supplementary Information [file 41467_2020_18591_MOESM1_ESM.pdf]

Trait Phenomenological Control Predicts Experience of Mirror Synaesthesia and the Rubber Hand Illusion

Lush et al

Supplementary Discussion

We reviewed the subjective report measures employed in the 20 most highly cited RHI studies, with counts ranging from 113 to 2895 citations per paper (returned by Google Scholar 16/01/2019). None reported the difference between reports in synchronous and asynchronous conditions as a measure of the rubber hand illusion (the method we employed for preregistered analyses). The majority tested for a difference between asynchronous and synchronous conditions in subjective response and then reported subjective responses in the synchronous condition as the measure of interest[1–17]. One paper [18] did not report a difference between synchronous and asynchronous conditions in this measure and a further two studies did not include an asynchronous condition[19,20]. Therefore the difference between subjective responses in synchronous and asynchronous conditions does not reflect common practice in measuring the rubber hand illusion.

We also reviewed the 20 most cited rubber hand papers which report proprioceptive drift results (with between 2895 and 117 citations). Of these, seven employed the difference between synchronous and asynchronous inductions as a measure of interest for proprioceptive drift[1,5,6,11,21–23] while 13 report instead the magnitude of drift following synchronous induction[4,9,10,14,17,18,24–30]. Proprioceptive drift is also most commonly measured by the magnitude of effect following synchronous induction. We therefore also conducted exploratory analyses on the magnitude of proprioceptive drift to allow comparison between these data and studies which report this measure.

Supplementary Method

Synchrony instruction condition: You will experience the illusion most strongly when the brush stroke you can feel on your own hand occurs in synchrony with the brushstroke you can see on the rubber hand. The illusion occurs because the relative timing of visual and tactile information influences whether or not the information is perceived as arising from a common source. The effect will therefore be induced when the brush strokes on the rubber hand and on your own hand are performed simultaneously.

Asynchrony instruction condition: Due to perceptual lag you will experience the illusion most strongly when the brush stroke on your own hand follows shortly after that on the rubber hand rather than occurring simultaneously. This effect is consistent with the neural delays involved in integrating visual and tactile stimuli. Researchers have found a 1/3 of a second to be the optimal delay, which we will approximately reproduce here in order to generate the maximum effect.

Supplementary Tables

		Instruction Condition			
Brushing condition	Measure	Synchrony (n = 114)	Asynchrony (n = 115)	None (control) (n = 124)	Combined (n = 353)
Synchrony					
	S1-3	*b* = .55 (.16), [.24, .86]	*b* = .65 (.18), [.30, 1.01]	*b* = .50 (.21), [.09, .90]	*b* = .57 (.10), [.37, .77]
	S4	*b* = .82 (.21), [.40, 1.24]	b = .95 (.24), [.49, 1.42]	*b* = .73 (.24), [.25, 1.20]	*b* = .83 (.13) [.57, 1.09]
	PD/cm	*b* = .49 (.40), [-.31, 1.29]	*b* = .009 (.37), [-.072, .74]	*b* = 1.06 (.32), [42, 1.70]	*b* = .58 (.21), [.17, .98]
Asynchrony					
	S1-3	b = .87 (.17), [.53, 1.21]	*b* = .61 (.21), [.20, 1.02]	*b* = .50 (.21), [.09, .91]	*b* = .70 (.11), [.48, .91]
	S4	*b* =.88 (.21), [.47, 1.29]	*b* = .95 (.24), [.49, 1.42]	*b* = .78 (.22), [.35, 1.20]	*b* = .87 (.12), [.63, [1.11]
	PD/cm	*b* =.08 (.35), [-.31, 1.29]	*b* = -.05 (.37), [-.78, .69]	*b* = .42 (.28), [-.01, .98]	*b* = .02 (.25), [-.48, .52]
Sum					
	S1-3	*b* =1.42 (.28), [.86, 1.98]	*b* = 1.26 (.31), [.65, 1.88]	*b* = .99 (.34), [.32, 1.67]	*b* = 1.25 (.18), [.90, 1.60]
	S4	*b* = 1.70, (.39), [.92, 2.46]	*b* = 1.89 (.43), [1.04, 2.73]	*b* = .73 (.24), [.25, 1.20]	*b* = 1.16 (.18), [.80, 1.52]
	PD/cm	*b* =.58 (.64), [-.69, 1.84]	*b* = -.037 (.66), [-1.35, 1.28]	*b* = 1.48 (.50), [.50, 2.45]	*b* = .83 (.34), [.17, 1.49]

Supplementary Table 1. Rubber hand illusion measures on subjective hypnotisability score (0-5) regression slopes (SE), [95% CI]

Supplementary references

1. Botvinick, M. & Cohen, J. Rubber hands 'feel' touch that eyes see. *Nature* **391**, 756–756 (1998).
2. Ehrsson, H. H. That's My Hand! Activity in Premotor Cortex Reflects Feeling of Ownership of a Limb. *Science* **305**, 875–877 (2004).
3. Ehrsson, H. H., Wiech, K., Weiskopf, N., Dolan, R. J. & Passingham, R. E. Threatening a rubber hand that you feel is yours elicits a cortical anxiety response. *Proc. Natl. Acad. Sci.* **104**, 9828–9833 (2007).
4. Kalckert, A. & Ehrsson, H. H. Moving a Rubber Hand that Feels Like Your Own: A Dissociation of Ownership and Agency. *Front. Hum. Neurosci.* **6**, (2012).
5. Rohde, M., Di Luca, M. & Ernst, M. O. The Rubber Hand Illusion: Feeling of Ownership and Proprioceptive Drift Do Not Go Hand in Hand. *PLoS ONE* **6**, e21659 (2011).
6. Suzuki, K., Garfinkel, S. N., Critchley, H. D. & Seth, A. K. Multisensory integration across exteroceptive and interoceptive domains modulates self-experience in the rubber-hand illusion. *Neuropsychologia* **51**, 2909–2917 (2013).
7. Hohwy, J. & Paton, B. Explaining Away the Body: Experiences of Supernaturally Caused Touch and Touch on Non-Hand Objects within the Rubber Hand Illusion. *PLoS ONE* **5**, e9416 (2010).
8. Dummer, T., Picot-Annand, A., Neal, T. & Moore, C. Movement and the Rubber Hand Illusion. *Perception* **38**, 271–280 (2009).
9. Thakkar, K. N., Nichols, H. S., McIntosh, L. G. & Park, S. Disturbances in Body Ownership in Schizophrenia: Evidence from the Rubber Hand Illusion and Case Study of a Spontaneous Out-of-Body Experience. *PLoS ONE* **6**, e27089 (2011).

10. Slater, M. Towards a digital body: The virtual arm illusion. *Front. Hum. Neurosci.* **2**, (2008).
11. Tsakiris, M., Carpenter, L., James, D. & Fotopoulou, A. Hands only illusion: multisensory integration elicits sense of ownership for body parts but not for non-corporeal objects. *Exp. Brain Res.* **204**, 343–352 (2010).
12. Moseley, G. L. *et al.* Psychologically induced cooling of a specific body part caused by the illusory ownership of an artificial counterpart. *Proc. Natl. Acad. Sci.* **105**, 13169–13173 (2008).
13. Guterstam, A., Petkova, V. I. & Ehrsson, H. H. The Illusion of Owning a Third Arm. *PLoS ONE* **6**, e17208 (2011).
14. Eshkevari, E., Rieger, E., Longo, M. R., Haggard, P. & Treasure, J. Increased plasticity of the bodily self in eating disorders. *Psychol. Med.* **42**, 819–828 (2012).
15. Newport, R., Pearce, R. & Preston, C. Fake hands in action: embodiment and control of supernumerary limbs. *Exp. Brain Res.* **204**, 385–395 (2010).
16. Kammers, M. P. M., de Vignemont, F., Verhagen, L. & Dijkerman, H. C. The rubber hand illusion in action. *Neuropsychologia* **47**, 204–211 (2009).
17. Durgin, F. H., Evans, L., Dunphy, N., Klostermann, S. & Simmons, K. Rubber Hands Feel the Touch of Light. *Psychol. Sci.* **18**, 152–157 (2007).
18. Ehrsson, H. H. Touching a Rubber Hand: Feeling of Body Ownership Is Associated with Activity in Multisensory Brain Areas. *J. Neurosci.* **25**, 10564–10573 (2005).
19. Peled, A., Ritsner, M., Hirschmann, S., Geva, A. B. & Modai, I. Touch feel illusion in schizophrenic patients. *Biol. Psychiatry* **48**, 1105–1108 (2000).
20. Peled, A., Pressman, A., Geva, A. B. & Modai, I. Somatosensory evoked potentials during a rubber-hand illusion in schizophrenia. *Schizophr. Res.* **64**, 157–163 (2003).

21. Tsakiris, M. & Haggard, P. The Rubber Hand Illusion Revisited: Visuotactile Integration and Self-Attribution. *J. Exp. Psychol. Hum. Percept. Perform.* **31**, 80–91 (2005).

22. Costantini, M. & Haggard, P. The rubber hand illusion: Sensitivity and reference frame for body ownership. *Conscious. Cogn.* **16**, 229–240 (2007).

23. Brozzoli, C., Gentile, G. & Ehrsson, H. H. That's Near My Hand! Parietal and Premotor Coding of Hand-Centered Space Contributes to Localization and Self-Attribution of the Hand. *J. Neurosci.* **32**, 14573–14582 (2012).

24. Longo, M. R., Schüür, F., Kammers, M. P. M., Tsakiris, M. & Haggard, P. What is embodiment? A psychometric approach. *Cognition* **107**, 978–998 (2008).

25. Tsakiris, M., Jiménez, A. T.- & Costantini, M. Just a heartbeat away from one's body: interoceptive sensitivity predicts malleability of body-representations. *Proc. R. Soc. B Biol. Sci.* **278**, 2470–2476 (2011).

26. Ehrsson, H. H. *et al.* Upper limb amputees can be induced to experience a rubber hand as their own. *Brain* **131**, 3443–3452 (2008).

27. Tsakiris, M., Costantini, M. & Haggard, P. The role of the right temporo-parietal junction in maintaining a coherent sense of one's body. *Neuropsychologia* **46**, 3014–3018 (2008).

28. IJsselsteijn, W. A., de Kort, Y. A. W. & Haans, A. Is This *My* Hand I See Before Me? The Rubber Hand Illusion in Reality, Virtual Reality, and Mixed Reality. *Presence Teleoperators Virtual Environ.* **15**, 455–464 (2006).

29. Haans, A., Kaiser, F. G., Bouwhuis, D. G. & IJsselsteijn, W. A. Individual differences in the rubber-hand illusion: Predicting self-reports of people's personal experiences. *Acta Psychol. (Amst.)* **141**, 169–177 (2012).

30. Shimada, S., Fukuda, K. & Hiraki, K. Rubber Hand Illusion under Delayed Visual Feedback. *PLoS ONE* **4**, e6185 (2009).

31. Grice-Jackson, T., Critchley, H. D., Banissy, M. J. & Ward, J. Common and distinct neural mechanisms associated with the conscious experience of vicarious pain. *Cortex* **94**, 152–163 (2017).

32. Ward, J., Schnakenberg, P. & Banissy, M. J. The relationship between mirror-touch synaesthesia and empathy: New evidence and a new screening tool. *Cogn. Neuropsychol.* **35**, 314–332 (2018).

33. Lush, P., Moga, G., McLatchie, N. & Dienes, Z. The Sussex-Waterloo Scale of Hypnotizability (SWASH): measuring capacity for altering conscious experience. *Neurosci. Conscious.* **2018**, (2018).

34. Bowers, K. The Waterloo-Stanford Group C (WSGC) Scale of Hypnotic Susceptibility: Normative and Comparative Data. *Int. J. Clin. Exp. Hypn.* **41**, 35–46 (1993).
