# Supplementary material for: Trait phenomenological control predicts experience of mirror synaesthesia and the rubber hand illusion
Source: Nat Commun. 2020 Sep 25;11:4853. doi: 10.1038/s41467-020-18591-6 (PMC7519080; doi:10.1038/s41467-020-18591-6)
Supplement: Supplementary file 3 — Reporting Summary [file 41467_2020_18591_MOESM3_ESM.pdf]

nature research

Corresponding author(s): Peter Lush

Last updated by author(s): Aug 18, 2020

Reporting Summary

Nature Research wishes to improve the reproducibility of the work that we publish. This form provides structure for consistency and transparency in reporting. For further information on Nature Research policies, see our Editorial Policies and the Editorial Policy Checklist.

Statistics

For all statistical analyses, confirm that the following items are present in the figure legend, table legend, main text, or Methods section.

n/a	Confirmed	
☐	☒	The exact sample size (*n*) for each experimental group/condition, given as a discrete number and unit of measurement
☐	☒	A statement on whether measurements were taken from distinct samples or whether the same sample was measured repeatedly
☐	☒	The statistical test(s) used AND whether they are one- or two-sided<br>*Only common tests should be described solely by name; describe more complex techniques in the Methods section.*
☒	☐	A description of all covariates tested
☐	☒	A description of any assumptions or corrections, such as tests of normality and adjustment for multiple comparisons
☐	☒	A full description of the statistical parameters including central tendency (e.g. means) or other basic estimates (e.g. regression coefficient) AND variation (e.g. standard deviation) or associated estimates of uncertainty (e.g. confidence intervals)
☐	☒	For null hypothesis testing, the test statistic (e.g. *F*, *t*, *r*) with confidence intervals, effect sizes, degrees of freedom and *P* value noted<br>*Give P values as exact values whenever suitable.*
☐	☒	For Bayesian analysis, information on the choice of priors and Markov chain Monte Carlo settings
☒	☐	For hierarchical and complex designs, identification of the appropriate level for tests and full reporting of outcomes
☐	☒	Estimates of effect sizes (e.g. Cohen's *d*, Pearson's *r*), indicating how they were calculated

Our web collection on statistics for biologists contains articles on many of the points above.

Software and code

Policy information about availability of computer code

Data collection	Custom software for computer delivery of SWASH hypnotisability screening. Available at https://osf.io/huwxd/
Data analysis	JASP 0.9. 2. and Dienes' Bayes factor calculator at http://www.lifesci.sussex.ac.uk/home/Zoltan_Dienes/inference/Bayes.htm

For manuscripts utilizing custom algorithms or software that are central to the research but not yet described in published literature, software must be made available to editors and reviewers. We strongly encourage code deposition in a community repository (e.g. GitHub). See the Nature Research guidelines for submitting code & software for further information.

Data

Policy information about availability of data

All manuscripts must include a data availability statement. This statement should provide the following information, where applicable:

- Accession codes, unique identifiers, or web links for publicly available datasets
- A list of figures that have associated raw data
- A description of any restrictions on data availability

The data that support the findings of this study are available at https://osf.io/huwxd/. All figures have associated raw data. There are no restrictions on data availability.

Field-specific reporting

Behavioural & social sciences study design

All studies must disclose on these points even when the disclosure is negative.

Study description	Two correlational studies investigating relationships between imaginative suggestibility in a hypnotic context and measures of embodiment. The purpose of these studies is to test predictions of the theory that demand characteristics can drive experience in psychological experiments.
Research sample	The research samples for both studies were drawn from psychology undergraduates. The studies investigate relationships between response to direct imaginative suggestion and established measures of experiential change. The investigated effects are commonly measured in psychology undergraduate students. We consider the samples employed are likely to be representative of participants in embodiment studies. For study Ia (vicarious pain), we analysed data from 404 participants (mean age= 19.1, SD= 2.4, 331 females, 73 males). For study Ib (mirror touch), we analysed data from 154 participants (mean age= 19.9, SD= 3.9, 133 females, 21 males). For study 2, data from 353 participants were analysed (mean age= 19.0, SD= 1.3, 276 females, 75 males, 2 other/not disclosed). Convenience samples were employed. For study 1 existing databases were combined, with all participants matched across databases included in analyses. For study 2, the entire 2018 cohort of 1st year Psychology were tested in laboratory practical sessions. We analysed Bayes factors, for which the data were shown to be sensitive.
Sampling strategy	The sample was the entire 1st year Psychology undergraduate cohort at University of Sussex. The sample size was therefore dictated by the Psychology intake for 2018. All inferences are based on Bayesian analysis, so predetermination of sample size is not relevant. Because Bayesian analyses are sensitive, sample sizes were adequate.
Data collection	Study 1 employed existing datasets. Therefore, researchers collecting the data were necessarily blind to hypotheses. For study 2, hypnotisability screening was conducted by computer. Participants listened to a pre-recorded script and responded using a keyboard. The rubber hand illusion procedure was recorded using pen and paper. Study 2 took place during lab sessions for psychology undergraduates. Approximately 30 students, 2 teaching assistants and 6 experimenters were present during these sessions. Researchers were blind to experimental conditions but were not blind to study hypothesis.
Timing	Data were collected between 15th and 19th October, 2018.
Data exclusions	There were no exclusions for Study 1. For Study 2, 30 participants were excluded due to a computer error during SWASH hypnotisability screening .
Non-participation	2 students declined participation because they did not want to take part in a hypnosis screening.
Randomization	Participants were not allocated into groups for study 1. Although the main findings are correlational, each effect comes from controlled experiments, and there is no competitor theory to ours that specifies a mediating variable that may undermine our conclusions. For study 2, participants were randomly allocated.

Reporting for specific materials, systems and methods

We require information from authors about some types of materials, experimental systems and methods used in many studies. Here, indicate whether each material, system or method listed is relevant to your study. If you are not sure if a list item applies to your research, read the appropriate section before selecting a response.

Materials & experimental systems

n/a	Involved in the study	
☒	☐	Antibodies
☒	☐	Eukaryotic cell lines
☒	☐	Palaeontology and archaeology
☒	☐	Animals and other organisms
☐	☒	Human research participants
☒	☐	Clinical data
☒	☐	Dual use research of concern

Methods

n/a	Involved in the study	
☒	☐	ChIP-seq
☒	☐	Flow cytometry
☒	☐	MRI-based neuroimaging

Human research participants

Policy information about studies involving human research participants

Population characteristics	See above
Recruitment	All participants were 1st year psychology undergraduates participating as part of a core module. The number of participants who refused to participate because of reluctance to take part in a hypnosis screening was to small to bias results.
Ethics oversight	This study was approved by the University of Sussex Sciences & Technology Cross-Schools Research Ethics Committee

Note that full information on the approval of the study protocol must also be provided in the manuscript.
